# Bioenergetics of life, disease and death phenomena

**DOI:** 10.1007/s12064-018-0266-5

**Published:** 2018-07-10

**Authors:** Andrzej Kasperski, Renata Kasperska

**Affiliations:** 1 Department of Biotechnology, Faculty of Biological Sciences, University of Zielona Gora, ul. Szafrana 1, 65-516 Zielona Gora, Poland; 2 Institute of Occupational Safety Engineering and Work Science, University of Zielona Gora, ul. Szafrana 4, 65-516 Zielona Gora, Poland

**Keywords:** Cancer attractors, Cancer development, Cancer transformation, Crabtree effect, Mitochondria, Warburg effect

## Abstract

In this article, some new aspects of unified cell bioenergetics are presented. From the perspective of unified cell bioenergetics certain subsequent stages of cancer development, from initiation stage, through transformation to metastasis, are analyzed. Here we show that after transformation, cancer cells are permanently exposed to reactive oxygen species, that causes continual random DNA mutations and as a result genome and chromosomal destabilizations. The modern cancer attractor hypothesis has been extended in explaining cancer development. Discussion is conducted in light of current cancerogenesis research, including bioenergetic cancer initiation, the somatic mutation theory and the tissue organization field theory. In the article reasons complicating the discovery of patterns of cancer genome changes and cancer evolution are presented. In addition certain cancer therapeutic aspects are given attention to.

## Introduction

The unified cell bioenergetics (UCB) is an extension of integrated cell bioenergetics and allows for the interpretation of several main cell bioenergetic effects, that can occur during the metabolism of eukaryotic cells, inclusive of Pasteur’s, Crabtree, Kluyver and glucose effects (Kasperski 2008; Kasperski and Kasperska 2013). According to UCB the occurrence of cellular bioenergetic effects and certain diseases are dependent on the intramitochondrial NAHD level. Mitochondria function like small intracellular factories converting compounds into energy. Places of energy production are usually a boon, but they may also pose threats. Similarly mitochondria (powerhouses of the cell) essential for life, have a negative side if damaged resulting in the release of reactive oxygen species (ROS) into the rest of the cell (Wei et al. 2017). A moderate ROS level may affect a number of cell biological processes through transcriptional regulation that include processes that have a direct impact on transcriptional regulation causing the modification of protein/enzyme molecules (i.e. TRX, AKt, PTEN, Bcl-2) involved in cell proliferation, transformation or survival, and other processes that have an indirect impact through the transcription factors (NF-κB, P53, Nrf-2, and HIF) (Trachootham et al. 2008a). A high ROS level can result in severe oxidative damage to the biolipid membranes, DNA, and proteins which may lead to cancer transformation and development (Trachootham et al. 2009; Heng 2016). Cancer cells have an altered energy metabolism which is substantially different than that of normal cells (Warburg 1956b; Gonzalez et al. 2012). This altered metabolism was first observed by Warburg (1930, 1956a, b). The Warburg effect discloses that cancer cells use fermentation even in the presence of oxygen. Cancer cell metabolism is characterized by limited energy metabolism due largely to glycolysis (this phenomenon is termed specifically ‘aerobic glycolysis’) characterized by an increase in glucose uptake and consumption, a decrease in oxidative phosphorylation (OXPHOS), and the production of lactate (Warburg 1956b; Hanahan and Weinberg 2011). As a result, there is a predominance of glycolysis and Cori cycle in cancer cells, this means that the cancer cells can reprogram their glucose metabolism (Warburg 1956b; Bustamante et al. 1981; Moreno-Sánchez et al. 2007). Another metabolic alteration in cancer cells is increased glutamine metabolism and altered lipid metabolism. Glucose and glutamine interact synergistically to drive cancer cell metabolism (Swinnen et al. 2006; Seyfried and Shelton 2010; Seyfried 2011; De Berardinis and Thompson 2012; Santos and Schulze 2012). One of the newest discoveries is possible activation of thymidine catabolism (observed in human gastric cancer), which can supply a carbon source in the glycolytic pathway and contribute to cell survival under conditions of nutrient deprivation (Tabata et al. 2017). The cancer hallmarks include: sustained proliferative signaling, evasion of growth suppressors, resistance to cell death, enablement of replicative immortality, energy metabolism list-reprogramming, evasion of immune destruction, inducement of angiogenesis, and the activation of invasion and metastasis. Genome instability (GIN) and chromosomal instability (CIN) and tumor promoting inflammation underlie the foregoing hallmarks (Zetter 1998; Hanahan and Weinberg 2011; Heng et al. 2013; Baker 2015; Goodson et al. 2015). Moreover, tumors contain a repertoire of recruited cells that contribute to the acquisition of the aforementioned hallmark traits by creating a ‘tumor microenvironment’ (Hanahan and Weinberg 2011). Most cancers are related to environmental, lifestyle or behavioral exposures which lead to bioenergetic destabilization (Szent-Györgyi 1973; Anand et al. 2008; Weiderpass 2010; Gonzalez et al. 2012; Baker 2015; Gonzalez et al. 2016). To date, an unequivocal explanation of cancer initialization has not been agreed upon. After cancer transformation, cancer development results in mutations numbering from about 10 to tens of thousands and chromosomal alterations (Greaves and Maley 2012; Stevens et al. 2013; Horne et al. 2015b). Occasionally the number of mutations can be larger than 100 thousands (Stratton 2011). A great deal of genetic heterogeneity in cancers is due to CIN (Heng et al. 2013). Cancer progression is driven by a small number of functionally important driver mutations. The rest of mutations are called ‘passengers’ (Greenman et al. 2007). Passenger mutations occur randomly and for this reason differ in all patients. Driver mutations in cancers of the same type are also typically different (Kauffman 1971). During cancer evolution, multiple variations inherited during cancer progression occur, and selection from this heterogeneity allows the obtainment of cancer cell advantage (Horne et al. 2015a). Cancer evolution can be considered stepwise, as a special case of the Darwinian process, with the accumulation of gene mutations during reiterative clonal expansion, intricate dynamic and highly variable patterns of genetic diversity, and clonal selection (Cairns 1975; Greaves and Maley 2012; Horne et al. 2013, 2015a). Nowadays, cancer genome sequencing projects have provided vast amounts of diverse, conflicting genomic data, which can be a considerable challenge for comprehension (Baker 2015; Horne et al. 2015a). Recent research has shown curiously that cancer cell genomes contain approximately about 10–200 mutations of a great variability, not found in wild type tissue. In view of the foregoing, the question of the true pattern of cancer evolution continues to remain the main challenge in understanding the mechanism of cancer evolution (Horne et al. 2015a). Since each cancer is different, with its own dynamic and variable genome, a new evolutionary approach and fresh ideas are required to unify practical observations (Horne et al. 2015a). The idea of ‘cancer attractors’, first suggested by Stuart Kauffman, has only been recently refreshed by experimental support genomic technologies (Kauffman 1971; Huang et al. 2009). Kauffman’s idea explains how gene regulatory networks ensure the ability to produce a variety of stable, discretely distinct cell phenotypes (Huang et al. 2009; Huang and Kauffman 2012). Cancer genome can be constituted as a complex network of mutually regulating genes (Greaves and Maley 2012). This network can lose stability due to GIN and CIN and can also, under certain conditions, produce hundreds of stable equilibrium states termed as attractors (Kauffman 1969a, b). Stable states depend on gene expression profiles associated with each cell type (Kauffman 1969a, 1993; Huang et al. 2009).

The article is organized as follows: firstly the methods and theoretical bases are listed, including description of the unified cell bioenergetics (UCB) and positive feedback for ATP (fATP). Secondly, in view of UCB various aspects of cellular bioenergetics are presented, with particular attention given to the fATP disturbances. Lastly, research conclusions are presented.

## Methods and theoretical basis

The results presented in this article are based on unified cell bioenergetics (UCB) (Kasperski 2008; Kasperski and Kasperska 2013). In accordance with UCB, the direct cause of metabolism regulation is positive feedback for ATP (fATP). ATP is the performer of metabolic regulation causing fATP, the local negative regulation of glycolysis, and the tricarboxylic acid (TCA) cycle (Krebs cycle). Because mitochondria are charged with NADH during the TCA cycle, and NADH is discharged from mitochondria in the electron transport chain (ETC), disturbances of fATP can change the amount of intramitochondrial high energy molecules (mtNADH and mtFADH_2_) (Kasperski 2008; Kasperski and Kasperska 2013).

**Fig. 1:**
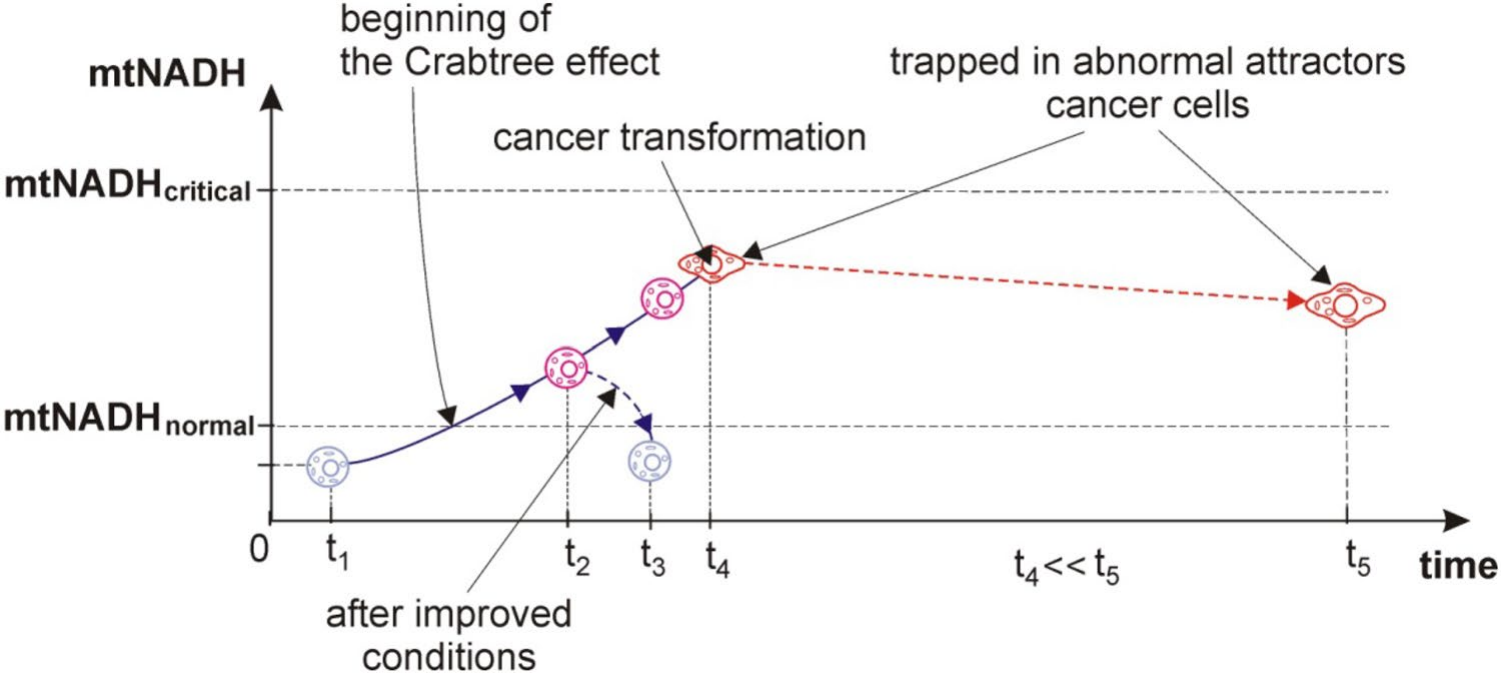
The level of intramitochondrial NADH (mtNADH) in a healthy cell (at *t*_1_), after beginning of disease (at *t*_2_), and after cancer transformation (at *t*_4_). Return to health (trajectory *t*_2_ → *t*_3_) by discharging mitochondria from an excessive amount of high energy molecules (especially mtNADH) is possible up until cancer transformation occurs. After cancer transformation the discharge of mitochondria from an excessive amount of high energy molecules can transpire very slowly (trajectory *t*_4_ → *t*_5_)—see Remarks 2, 5. In this case, cells remain trapped in abnormal attractors

According to UCB each cell contains specific mtNADH_normal_ and mtNADH_critical_ levels (where mtNADH_critical_ > mtNADH_normal_, Fig. 1) (Kasperski 2008; Kasperski and Kasperska 2013). Increase of the intramitochondrial NADH (mtNADH) to a mtNADH_critical_ level causes a gradual inhibition of the TCA cycle; exceeding this level results in full blockage of the TCA cycle. Additionally, exceeding the mtNADH_normal_ level while the increasing intramitochondrial NADH, starts and then stimulates fermentation. Decrease of the mtNADH to the mtNADH_normal_ level causes gradual inhibition of fermentation; exceeding this level causes full fermentation blockage. Moreover, exceeding the mtNADH_critical_ level while decreasing mtNADH, starts and then stimulates the TCA cycle (Kasperski 2008; Kasperski and Kasperska 2013). An increase of the amount of intramitochondrial high energy molecules (i.e. when mtNADH > mtNADH_normal_, Fig. 1) causes an occurrence of the reversible Crabtree effect, i.e. fermentation in good aerobic conditions (Kasperski 2008).

### Power of positive feedback for ATP

Mitochondria as biosynthetic and bioenergetic organelles play an important role in producing metabolites and ATP, byproducts of the Krebs cycle (Martínez-Reyes et al. 2016). In an adult human being, mitochondria synthesize about 40 kg of ATP per day (consuming about 400 l of O_2_ per day), so it is easy to imagine the deleterious consequences of malfunctioning OXPHOS (Lyamzaev et al. 2008). For example the ATP storage alteration is found in several human disorders, including cancer (for this reason, measurement of ATP levels is utilized to evaluate cancer growth and to determine cancer chemosensitivity) (Sevin et al. 1988; Bradbury et al. 2000; Patergnani et al. 2014). Moreover, mitochondria are charged with NADH during the Krebs cycle, then discharged with NADH in the electron transport chain (ETC), which converts NADH to NAD. After using 2 ATP molecules for activation of 1 glucose molecule, 6 NADH molecules and 2 FADH_2_ molecules in mitochondria are created. This means that destabilization/dysregulation of fATP can also cause an accumulation of intramitochondrial NADH (mtNADH) (Kasperski 2008). Accumulation of mtNADH can occur because of mitochondrial inner membrane impermeability to NADH (Overkamp et al. 2000; Bakker et al. 2001). mtNADH is accumulated when the rate of mitochondria charging with NADH (*V*_(+)NADH_) exceeds the rate of NADH discharging from mitochondria (*V*_(−)NADH_). In accordance with unified cell bioenergetics, the interrelationships between *V*_(+)NADH_ and the *V*_(−)NADH_ may determine the occurrence of effects observed in the life of eukaryotic cells, for example, an excessive amount of mtNADH causes cells to switch to oxido-fermentative metabolism (i.e. the reversible Crabtree effect, Fig. 1) (Kasperski 2008).

In good aerobic conditions, the ATP yield for glucose oxidation is dependent on the efficiency of ATP synthase in converting proton gradient into energy stored in ATP. In order to exhibit the power of fATP, a net yield equal to 36 ATP (obtained using 2 ATP molecules for activation of 1 glucose molecule) was assumed (Nelson et al. 2008; Szigeti et al. 2017). Additionally, it was assumed that 1/5 of gained ATP is used in the activation of glucose, 4/5 of gained ATP is used for life processes, and that cellular control of energetic gain has been disrupted resulting in occurrences fATP disturbance. The disturbance causes *V*_(+)NADH_ > *V*_(−)NADH_ with assumed consequences of 1 NADH molecule to accumulate in mitochondria after each Krebs cycle rotation. Under such assumptions, the energetic gain after the first glucose oxidation cycle is equal to 30 ATP molecules [i.e. 36 ATP - 2 × 3 ATP molecules, because theoretically in ETC, 1 NADH molecule gives 3 ATP molecules (Nielsen et al. 2003)]. Then, 1/5 of gained ATP returns and is used for the next activation of glucose, so that the energetic gain after the second glucose oxidation cycle is equal to 3 × 30 ATP molecules—i.e. feedback for ATP is positive. Subsequently next 1/5 of gained ATP returns and is used for glucose consequent activation, so the energetic gain after the third glucose oxidation cycle is equal to 3 × 3 × 30 ATP molecules. After n’th oxidation cycle, the energetic gain is equal to 3^(*n*−1)^ × 30 ATP molecules. The energetic gain and amount of accumulated mtNADH, in successive glucose oxidation cycles, are presented in Table 1. From Table 1 it can be observed that short term destabilization/dysregulation of fATP can cause serious cellular bioenergetic problems along with a huge accumulation of NADH in mitochondria.

**Table 1:** The power of positive feedback for ATP demonstrated by energetic gain and amount of accumulated mtNADH in successive glucose oxidation cycles

Glucose oxidation cycle	ATP molecule gain	Accumulated mtNADH molecules
1	30	2
2	90	6
3	270	18
4	810	54
5	2430	162
…	…	…
10	590,490	39,366
…	…	…
20	34,867,844,010	2,324,522,934
…	…	…
30	2,058,911,320,946,490	137,260,754,729,766

## Results and discussion

In this section we would like to identify destabilization/dysregulation of positive feedback for ATP (fATP) as a possible underlying reason for cancer. Destabilization/dysregulation of fATP can induce mitochondria charging with high energy molecules to become higher than the rate of mitochondria discharge. In these conditions an accumulation of high energy molecules occurs, especially the accumulation of intramitochondrial NADH (mtNADH) resulting in an increase in the rate of ROS formation (Kushnareva et al. 2002; Cortassa et al. 2004; Kasperski 2008; Kasperski and Kasperska 2013).

### The manner in which positive feedback for ATP destabilization/dysregulation can lead to cancer transformation

In accordance with unified cell bioenergetics, destabilization/dysregulation of positive feedback for ATP (fATP) can cause an increase in the amount of intramitochondrial high energy molecules (especially mtNADH), i.e. mitochondria are overenergized (Kasperski 2008; Kasperski and Kasperska 2013).

#### *Remark 1*

It is known that cancer cells have higher NADH levels than healthy cells (Pelicano et al. 2006; Yu and Heikal 2009). An increase in the concentration of intramitochondrial NADH plus an imbalance of flavin coenzymes functionally halts the NADH shuttle in a retrograde fashion, with the result that the increased level of NADH in the mitochondria spreads on to the cytoplasm (Noda et al. 2002).

**Fig. 2:**
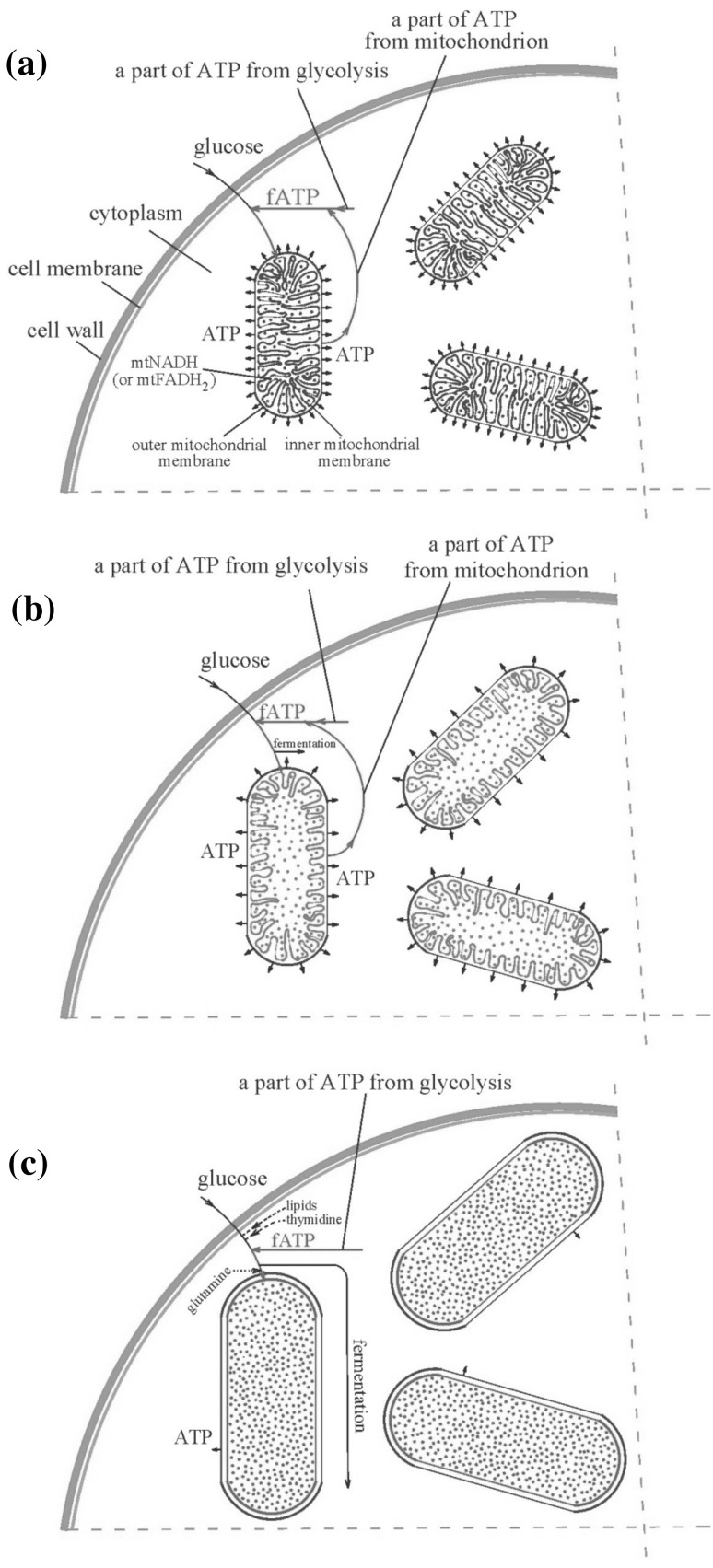
The positive feedback for ATP (fATP) in **a** healthy cell, **b** at disease outset after deep penetration into oxido-fermentative metabolism (due to the reversible Crabtree effect), **c** after cancer transformation

As previously stated, NADH accumulation causes an increase in ROS rate formation. Under the assumption that most electron donors are in a nonreducing state, the rate of ROS formation increases exponentially with NADH concentration (Cortassa et al. 2004). Increased ROS levels in cancer cells compared to normal cells have been reported by other researchers (Lu et al. 2007; Trachootham et al. 2008b, 2009; Weinberg and Chandel 2009; Wen et al. 2013; Yang et al. 2013). Increased levels of ROS may lead to DNA damage and to the direct activation of signaling networks, promoting tumorigenesis and metastasis (Fogg et al. 2011). When the ROS level is high, the number of random DNA mutations increases (i.e. the number of passenger mutations multiply) (Liou and Storz 2010). Random mutations caused by ROS can also change the DNA fragments which code mechanisms responsible for monitoring genomic integrity (Liou and Storz 2010). A defect in the regulation of these mechanisms can cause genome (GIN) and chromosomal instability (CIN), predisposing the cell to cancer transformation (Roschke and Kirsch 2010; Fogg et al. 2011; Al-Sohaily et al. 2012; Yao and Dai 2014; Goodson et al. 2015; Langie et al. 2015). This is in accord with traditional research, which holds that stepwise accumulated gene mutations are the keys to cancer development (Heng 2016). A cell with GIN and CIN can undergo cancer transformation with cancer symptoms including Warburg effect symptoms (see “Introduction” section) (Yao and Dai 2014). This is a cell preprogrammed transformation mechanism (see “Preprogrammed cell transformations” section), initiated after severe DNA damage (Salmina et al. 2010; Erenpreisa 2014; Jang et al. 2015; Vazquez-Martin et al. 2016). The probability of a single cell with GIN and CIN undergoing preprogrammed transformation to a cancer cell is very slight (Calabrese and Shibata 2010). The occurrence of fATP in a healthy cell, at disease outset, and after cancer transformation, is presented in Fig. 2. Cancer transformation initiates also cancer cell creation multiple clones to increase survival probability (Cooper 2000). In addition, the Warburg effect leads to altered energy flow (see “Introduction” section). Cancer cells gain energy through highly intensive aerobic glycolysis, that ensures cell energetic requirements in the early stage of cancer development and inhibits overenergized mitochondria discharge (see Remark 2). This means that cell mitochondria remain charged after cancer transformation, i.e. the level of mtNADH remains high after transformation in accordance with Remark 1.

#### *Remark 2*

High-intensity ATP production is necessary to fulfill the strong energy cancer cell demand for permanent proliferation (Szigeti et al. 2017). The switch to aerobic glycolysis and the deactivation of OXPHOS begin rapid generation of ATP in the glycolysis-fermentation pathway (fermentation allows NAD restitution in the cytoplasm through the reduction of pyruvate) (Ratledge 1991). In these conditions using 2 ATP for glucose activation allows to obtainment of 4 ATP, with an energetic gain equal to 2 ATP (Nelson et al. 2008). This means that OXPHOS efficacy is about 18 times higher at the end due to the ATP amount. Owing to a possible fermentation rate 100 times quicker than the oxidative process, fermentation produces at the end about six times more ATP during a given period in comparison to OXPHOS (Larson 2004; Szigeti et al. 2017).

**Fig. 3:**
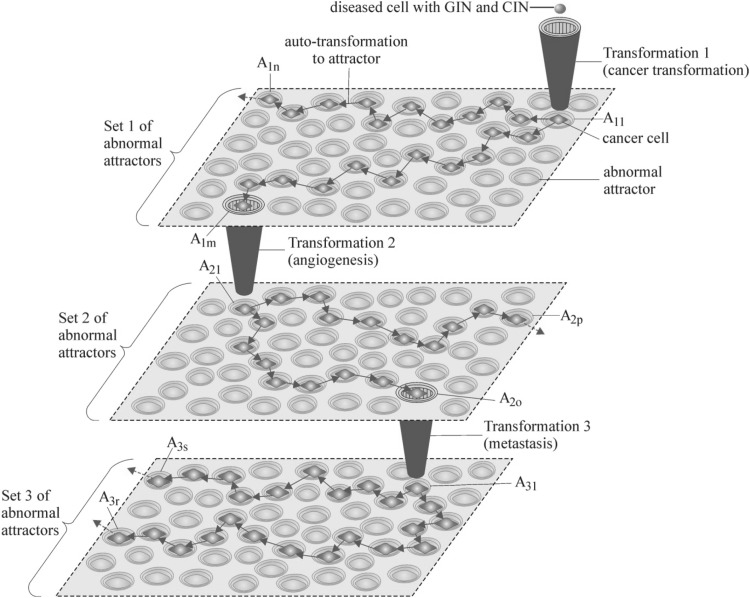
Preprogrammed cell transformations. Transformation 1 leads to set 1 of abnormal attractors featuring: sustained proliferative signaling, growth suppressor evasion, resistance to cell death, replicative immortality enablement, deregulated metabolism, and immune destruction evasion. Exemplary (*A*_11_ → *A*_1*m*_) trajectory leads to Transformation 2, and exemplary (*A*_11_ → *A*_1*n*_) trajectory does not lead to transformation. Transformation 2 leads to set 2 of abnormal attractors allowing cancer cells to obtain an additional feature of angiogenesis inducement. Exemplary (*A*_21_ → *A*_2*o*_) trajectory leads to Transformation 3, and exemplary (*A*_21_ → *A*_2*p*_) trajectory does not lead to transformation. Transformation 3 leads to set 3 of abnormal attractors allowing cancer cells to obtain additional invasion and metastasis activation features. Two exemplary trajectories (*A*_31_ → *A*_3*r*_) and (*A*_31_ → *A*_3*s*_) are presented at this level. After attaining abnormal attractors by clones, permanent exposition to ROS causes an accumulation of random DNA mutations resulting in genome instability (GIN) and chromosomal instability (CIN). GIN and CIN cause clones to leave attractors, and by way of auto-transformations to attractors, attain new abnormal attractors (i.e. clones permanently change attractors) or subsequent transformations (see Remarks 3, 4)

A high mtNADH level maintains a high level of ROS, that causes permanent random DNA mutations (i.e. the number of passenger mutations constantly increases), leading to subsequent GIN and CIN of the transformed cells. After this GIN and CIN occurrence, according to cancer attractor hypothesis, cell genomes undergo change to attain a steady state at ‘lowest energy state’ (Huang et al. 2009). This type of transformation is referred to as ‘auto-transformation to attractor’. In this way GIN and CIN lead to new abnormal attractors or to subsequent preprogrammed transformations. Each preprogrammed transformation leads to new sets of abnormal attractors (Fig. 3). It can be said that cancer clones are trapped in abnormal attractors (Huang et al. 2009). The trapped cancer clones remain exposed to ROS due to persistently elevated level of mtNADH (see Remark 1). The accumulation of random DNA mutations are the basis for consequent GIN and CIN, causing cells to leave current abnormal attractors and through genome stabilization are transferred to new abnormal attractors or undergo preprogrammed transformations (Fig. 3).

### Mitochondrial membrane potential as health, carcinogenesis and apoptosis indicator

Healthy cells maintain stable levels of mitochondrial membrane potential (MMP) and intracellular ATP, this stability is required for cell viability (Zamzami et al. 1995; Izyumov et al. 2004; Yaniv et al. 2010; Zorov et al. 2014). The optimal range of MMP for maximal ATP generation by OXPHOS is [136, 140] mV (Bagkos et al. 2014). MMP arises as a consequence of the pumping of protons out of the matrix (then, this potential is utilized during ATP synthesis) (Henry-Mowatt et al. 2004). In view of this and according to unified cell bioenergetics (UCB), the intramitochondrial NADH (mtNADH) accumulation occurring during cancer (see previous section) leads to proton concentration out of the matrix resulting in increased MMP, and also concentration of superoxide anions (as the main undesired by-product of mitochondrial oxidative phosphorylation) inside the matrix (Suski et al. 2012). Superoxide is the proximal mitochondrial reactive oxygen species (ROS) (Murphy 2009). These considerations demonstrate that increased MMP value is associated with increased ROS. There is research that corroborates increased MMP value with increased ROS production (Korshunov et al. 1997; Turrens 2003; Suski et al. 2012; Martínez-Reyes et al. 2016). It is known that generation of ROS increases exponentially with increased MMP, i.e. mitochondria produce more ROS when MMP is high (Liu and Huang 1996; Skulachev 1996; Korshunov et al. 1997; Starkov and Fiskum 2003). Moreover, ROS production dramatically increases when MMP is above 140 mV, i.e. when MMP is above the optimal MMP range for maximal ATP generation by OXPHOS (Korshunov et al. 1997; Bagkos et al. 2014). Increased ROS stress in cancer cells is correlated with aggressive tumors and poor prognosis, diagnosed by MMP measurement, i.e. increased aggressiveness and metastatic cancer occurring with elevated hyperpolarized MMP (Heerdt et al. 2005; Patel et al. 2007; Kumar et al. 2008). This can be exemplified by Neu4145 cancer cells, for which MMP is equal about 210 mV (Fantin et al. 2006). In sum, in view of UCB, cancer cells exhibit more hyperpolarised MMP compared to healthy cells. This correlates with other research findings, from which it can be stated that MMP can be considered to be a reliable carcinogenesis indicator (Heerdt et al. 2005; Fantin et al. 2006; Bonnet et al. 2007; Wen et al. 2013).

There is a phenomenon contrary to carcinogenesis, apoptosis (programmed cell death) which causes decreased MMP value (Gottlieb et al. 2003). In accordance with UCB, decrease in MMP is caused by decreasing positive feedback for ATP (fATP) (with resulting mtNADH decrease), resulting from cessation of vital cell functions. MMP decrease leads to matrix condensation and cytochrome c exposure to intermembrane space, facilitating release of cytochrome c and cell death proceeding from apoptotic insult (Gottlieb et al. 2003).

#### *Remark 3*

This part of the article can be summed up as:

- Cancer cells have higher NADH levels than healthy cells.
- Higher NADH levels cause higher rate of ROS generation. This phenomenon results in cancer cells exhibiting increased levels of ROS than normal cells.
- Higher levels of ROS generate random DNA mutations.
- Random DNA mutations caused by ROS can change the DNA fragments which code mechanisms responsible for monitoring genomic integrity and as a result lead to GIN and CIN.
- GIN and CIN predispose the cell to cancer transformation and cancer development.
- Higher mtNADH levels lead to increased MMP value. The more aggressive and metastatic the cancer, the more hyperpolarized MMP.

### Preprogrammed cell transformations

Higher levels of ROS may play a significant role in hallmark cancer acquisition (Hanahan and Weinberg 2000), immortalization and transformation (Behrend et al. 2003), cancer cell proliferation (Achanta et al. 2005), mitogenic signaling (Irani et al. 1997), cell survival and disruption of cell death signaling (Pervaiz and Clement 2004; Clerkin et al. 2008; Trachootham et al. 2008a), chemoresistance (Pervaiz and Clement 2004; Achanta et al. 2005), angiogenesis (Komatsu et al. 2008; Ushio-Fukai and Nakamura 2008), epithelial–mesenchymal transition and metastasis (Radisky et al. 2005; Ishikawa et al. 2008; Fogg et al. 2011)—more detail information is to be found in Gupta et al. 2012 and Yang et al. 2013. There are hundreds of possible abnormal attractors, that can be attained by cells during cancer development (Kauffman 1969a, b; Huang et al. 2009). In view of Remark 3, new attractors can be attained consequent to genome and chromosomal instabilities (resulting from random DNA mutations caused by ROS), through auto-transformations to attractors and also through preprogrammed transformations (Fig. 3).

#### *Remark 4*

Cancer development leads to the outgrowth of a clonally derived population of cancer cells. Cancer development continues as mutations occur within cancer population cells, with attained attractors dependent on gene expression profile associated with cancer cell type (see “Introduction” section). Some of these mutations can add selective advantages to the cells (Cooper 2000). Such advantages include for example accelerated growth and possible use of different carbon and energy sources (activation of glutamine, thymidine and altered lipid metabolism—see “Introduction” section). Cell descendants that have acquired these advantages will become dominant within the cancer population (clonal selection). Clonal selection participates cancer growth and malignancy (Cooper 2000).

Based on Brücher and Jamall work, three preprogrammed transformations can be distinguished (Brücher and Jamall 2014). Subsequent preprogrammed transformations usually occur when the cancer becomes sizeable and more diverse, but there can be exceptions, and all preprogrammed transformations can be concurrent (Bernards and Weinberg 2002; Weinberg 2008; Calabrese and Shibata 2010). Preprogrammed transformations lead to new sets of abnormal attractors, that extent the current cancer attractor hypothesis (Fig. 3).

#### *Transformation 1*

(Cancer transformation with cancer symptoms including Warburg effect symptoms—see “Introduction” section)—This transformation constitutes the input to set 1 of abnormal attractors (Fig. 3). Each abnormal attractor in set 1 exhibits features of: sustained proliferative signaling, growth suppressor evasion, resistance to cell death, replicative immortality enablement, deregulated metabolism, and immune destruction evasion.

#### *Transformation 2*

(Induction of angiogenesis)—This transformation constitutes the input to set 2 of abnormal attractors (Fig. 3), allowing cancer cells to obtain an additional angiogenesis inducement feature.

#### *Transformation 3*

(Activation of invasion and metastasis)—This transformation constitutes the input to set 3 of abnormal attractors (Fig. 3), allowing cancer cells to obtain additional invasion and metastasis activation features.

### Reasons causing positive feedback for ATP destabilization/dysregulation

In view of the considerations presented in foregoing sections we would like to point out that bioenergetic (Case 1) and genetic problems (Case 2), and disruption of interactions with adjacent tissue (Case 3) can lead to cancer. According to unified cell bioenergetics (UCB) all of the aforementioned reasons can cause the destabilization/dysregulation of positive feedback for ATP (fATP). Due to the charging of mitochondria with NADH during the TCA cycle, and the discharging of mitochondria from NADH in the electron transport chain (ETC), disturbances of fATP stimulating the TCA cycle and/or inhibiting ETC can engender accumulation of intramitochondrial high energy molecules (mtNADH and mtFADH_2_)—for more details, see in Kasperski 2008, and Kasperski and Kasperska 2013, where this mechanism is described in detail.

Case 1—bioenergetic problems.

- Cellular bioenergetic problems caused by deleterious living conditions (e.g. insalubrious environmental conditions, unhealthy life styles), for example cigarette smoke and/or environmental pollution inhibiting ETC, reduced oxygen concentration, excessive nutrient (sugar) concentration, continual stress, lack of sufficient exercise resulting in collection of poisoning metabolites, and long lasting infectious wounds can cause fATP destabilization/dysregulation leading to, in accordance with UCB, an overenergization of mitochondria filling them with high energy molecules (especially NADH). Case 1 consequences can be written thusly:where *V*_(+)NADH_—rate of mitochondria charging with NADH, *V*_(−)NADH_—rate of NADH discharging from mitochondria, mtNADH—intramitochondrial NADH, cytNADH—cytosolic NADH, LPR—lactate production rate, ROS—reactive oxygen species, GIN, CIN—genome and chromosomal instability.
  Lingusticial interpretation of Case 1 consequences: such (deleterious) living conditions can occur $\left( \mathop{\exists}\limits_{LivingCon}  \right)$, that cause fATP destabilization/dysregulation and, as a result, in accordance with UCB, the charging rate of mitochondria with NADH exceeds the NADH discharge rate from mitochondria (*V*_(+)*NADH*_ > *V*_(−)*NADH*_). Therefore, the amount of intramitochondrial and cytosol NADH increases (mtNADH $\uparrow\wedge$ cytNADH ↑)—see Remark 1. As a result, the amount of mtNADH exceeds the mtNADH_normal_ level (Fig. 1).
  Subsequently, resulting from higher NADH levels, symptoms auguring impending cancer transformation begin to appear, among others, increased lactate buildup (LPR ↑, i.e. the reversible Crabtree effect occurs) along with increased ROS generation (ROS ↑) causing an increase of random DNA mutations (*random DNA*_<*MUT*>_↑). These random DNA mutations can change the DNA fragments which code mechanisms responsible for monitoring genomic integrity causing GIN and CIN (see Remark 3). GIN and CIN predispose the cell to cancer transformation with cancer symptoms including Warburg effect symptoms (see “Introduction” section). After cancer transformation, GIN and CIN lead to cancer development (see Remark 3 and Fig. 3).

#### *Remark 5*

In view of UCB, the switch to aerobic glycolysis and deactivation of OXPHOS can halt of mtNADH increase after cancer transformation (Fig. 1). However, many cancers maintain active OXPHOS along with greater or less anaerobic ATP production (this phenomenon may be explained by differences in tumor size, hypoxia, and the sequence of activated oncogenes) (Jose et al. 2011).

- Case 2—genetic problems.
  DNA code mutations (especially mutations, which cause gene disruption responsible for ETC) can cause fATP destabilization/dysregulation leading to, in accordance with UCB, an overenergization of mitochondria filling them with high energy molecules (especially NADH). Case 2 consequences can be written thusly:
  Lingusticial interpretation of Case 2 consequences: in any living conditions $\left( {\mathop \forall \limits_{LivingCon} } \right)$, such specific DNA mutations (or mutation) can occur $\left( {\mathop \exists \limits_{{DNA_{ < MUT > } }} } \right)$, that cause fATP destabilization/dysregulation and, as a result, in accordance with UCB, the charging rate of mitochondria with NADH exceeds the NADH discharge rate from mitochondria (*V*_(+)*NADH*_ > *V*_(−)*NADH*_). Therefore, the amount of intramitochondrial and cytosol NADH increases (mtNADH $\uparrow\wedge$ cytNADH ↑)—see Remark 1. As a result, the amount of mtNADH exceeds the mtNADH_normal_ level (Fig. 1).
  Subsequently, as a result of higher NADH levels, symptoms auguring impending cancer transformation begin to appear, and then, after transformation, cancer symptoms occur (see lingusticial interpretation Case 1).

Occurrence of Case 2 is in accordance with the somatic mutation theory (SMT), which holds that cancer begins with a mutation that gives cells growth advantage, leading to clonal expansion and successive mutations followed by further clonal expansion (Baker 2015) (see Remark 4). The premises of SMT can be stated thusly: (a) cancer is derived from a single somatic cell that has accumulated multiple DNA mutations, (b) cancer is a disease of cell proliferation caused by mutations in genes that control proliferation and cell cycle (Greaves and Maley 2012; Sonnenschein et al. 2014; Baker 2015) (see Remark 3). In view of Case 2, cancer initiation can occur in any (i.e. also very salubrious) living conditions, but in accordance to Case 1 deleterious living conditions can amplify the cancer initiation process.

- Case 3—cell interaction disruption.
  Disruption of cell interactions with adjacent tissue can cause connection loss to external signals. This disruption can result in fATP destabilization/dysregulation because of possible lost of metabolism adaptation to ongoing environmental changes inside and outside the organism. As a result of this disruption, in accordance with UCB, an overenergization of mitochondria filling them with high energy molecules (especially NADH) can occur. Case 3 consequences can be written thusly:
  Lingusticial interpretation of Case 3 consequences: such disruption of cell interactions with adjacent tissue can occur $\left( {\mathop \exists \limits_{InterDis} } \right)$, that causes fATP destabilization/dysregulation and, as a result, in accordance with UCB, the charging rate of mitochondria with NADH exceeds the NADH discharge rate from mitochondria (*V*_(+)*NADH*_ > *V*_(−)*NADH*_). Therefore, the amount of intramitochondrial and cytosol NADH increases (mtNADH $\uparrow\wedge$ cytNADH ↑)—see Remark 1. As a result, the amount of mtNADH exceeds the mtNADH_normal_ level (Fig. 1).
  Subsequently, as a result of higher NADH levels, symptoms auguring impending cancer transformation begin to appear, and then, after transformation, cancer symptoms occur (see lingusticial interpretation Case 1).

Occurrence of Case 3 is in accordance with the tissue organization field theory (TOFT), that states that cancer arises from the disruption of interactions with adjacent tissue, which can be mediated by intercellular chemical signals, mechanical forces, and bioelectric changes (Baker 2015).

### Pattern of cancer mutations

In accordance with unified cell bioenergetics (UCB), pointed out previously in this article, cancer initiation can be considered as a random process (see “Reasons causing positive feedback for ATP destabilization/dysregulation” section), resulting from an accumulation of random DNA mutations engendered by ROS. This results in genome (GIN) and chromosomal instability (CIN), leading to cancer transformation, with cancer cells remaining exposed to ROS (see Remark 3). An excessive amount of ROS is maintained inside mitochondria (due to a substantial level of high energy molecules—see Remarks 1, 3), which can induce transformed cell genome (GIN) and chromosomal instability (CIN). GIN and CIN lead to new abnormal attractors (through auto-transformation to attractor) or can lead to subsequent preprogrammed transformations. Genome changes caused by auto-transformation to attractor allow the attainment of a steady state (‘lowest energy state’) in an ordered way, and for this reason, mutations caused by auto-transformations to attractors, can be classified as drivers. Requisite driver mutations, introduced by auto-transformation to attractor to attain new steady state, are dependent upon the specific gene expression profile associated with cancer cell type (see Remark 4) and existing driver and random passenger mutations (i.e. current state of the network)—this is why driver mutations in cancers of the same type are typically quite different. Considering the foregoing, ascertainment of cancer genome mutation patterns is very difficult. Conversely, it is not difficult to ascertain the reason for cancer dissimilarity and cancer genome diversity and ‘noisiness’.

### Medical aspects in view of unified cell bioenergetics

In accordance with unified cell bioenergetics (UCB), the medical consequences of destabilization/dysregulation of positive feedback for ATP, resulting in the overenergetization and overloading of mitochondria with high energy molecules, are:

- Cellular bioenergetic problems—an excessive amount of mtNADH leads to deep penetration into oxido-fermentative metabolism (i.e. occurrence of the reversible Crabtree effect, Fig. 1) and inhibition of the Krebs cycle. Moreover, the increased level of mtNADH spreads on to the cytoplasm (see Remark 1).
- Diabetes—an accumulation of NADH (halting the Krebs cycle and facilitating anaerobic glucose metabolism) in beta-cells is observed with diabetes (Noda et al. 2002).
- Apoptosis—higher NADH levels cause a higher rate of ROS generation, which can lead to severe oxidative damage to biolipid membranes, proteins and DNA (see Remark 3). Mitochondria normally initiate apoptosis when damage to the respiratory apparatus becomes critical (Wallace 2005).
- Cancer—cells that do not undergo apoptosis can be transformed into cancerous ones (see Remarks 1, 3).

In a sense, small bioenergetic problems are one of the first signals of an improper lifestyle. Apoptosis can also protect cells from cancer transformation (Roninson et al. 2001; Erenpreisa et al. 2017). It is known that apoptosis plays a fundamental role in animal development and tissue homeostasis, eliminating unwanted, abnormal, injured, or dangerous cells. Dysregulation of this process is associated with a variety of human diseases such as immunological and developmental disorders, neurodegeneration, and cancer (Jacobson et al. 1997; Fuchs and Steller 2011; Tang et al. 2012). After cancer transformation cells remain exposed to ROS influence because of a high level of mtNADH (see Remarks 1). In these conditions, GIN and CIN of transformed cells can occur (see Remark 3). GIN and CIN cause cancer development by leading to new abnormal attractors or to subsequent preprogrammed transformations (see Remarks 3, 4 and Fig. 3). In order to terminate this permanent phenomenon of changing abnormal attractors, cancer cell mitochondria should be discharged from an excessive amount of high energy molecules (in order to decrease ROS amount). This is consistent with other research findings that efforts should be directed towards eliminating the ‘noise’ of heterogeneity in cancer, pushing the genome of cancer cells to stability and followed with immunological system support (Heng et al. 2009; Alvergne et al. 2016). Discharging mitochondria from an excessive amount of high energy molecules however is somewhat of a challenge, because cancer cells gain considerable energy from intensive aerobic glycolysis, that inhibits overenergized mitochondria discharge (see Remark 2). For this reason, discussion should be undertaken as to whether a better solution would be to stop random DNA mutations by ROS (aiming to disallow GIN and CIN thereby decreasing cancer genetic heterogeneity) by destroying of cancer cell mitochondria. This research finding is in accordance with a recent Penn State University study, which found that the main antioxidant in green tea, epigallocatechin-3-gallate (EGCG), helps kill cancer cells by destroying cell mitochondria. Up until now tests have targeted pancreatic and oral cancers (Tao et al. 2015). Our finding is also consistent with opinion of another research group, which observed that removal of damaged mitochondria by autophagy is important for cellular health (Wei et al. 2017). Taking into account UCB, increased fresh air physical activity together with stress and sugar avoidance, in order to discharge mitochondria from an excessive amount of high energy molecules and maintenance of a mtNADH amount below mtNADH_normal_ level, along with stabilization of positive feedback for ATP may afford healthier life. These health recommendations are also supported by even additional studies finding cancer to be a disease requiring major lifestyle changes, highlighting extensive evidence suggesting physical exercise can reduce the incidence of various cancers (Anand et al. 2008; Weiderpass 2010; Draghi et al. 2017).

## Conclusions

In this article, selected aspects of unified cell bioenergetics (UCB) and positive feedback for ATP (fATP) have been presented. It has been pointed out that fATP drives life, but also can induce disease and death. It has been shown that a healthy life requires stabilized fATP, because the destabilization/dysregulation of fATP can bring about an accumulation of high energy molecules in mitochondria leading to health problem onset. In the article, we tried to point out, that cancer cells can be considered as casualties, among others, deleterious living conditions (see Case 1—bioenergetic problems) or misfortune (see Case 2—genetic problems) trapped, according to Kauffman’s idea, in abnormal attractors. Three reasons for cancer initiation at cellular level (i.e. bioenergetic and genetic problems and cell interaction disruption) have been presented based on UCB. Additionally, it has been pointed out that the three aforementioned causes can be interpreted universally using UCB. In accordance with UCB it has been shown that cancer initiation can be considered as a random process engendered by random DNA mutations effectuated by ROS. The study presents that cells after cancer transformation remain exposed to ROS driving cancer development (see Remarks 3, 4, 5 and Fig. 1) and leading to very complex genome mutation patterns. The presented findings may reveal certain prophylactic aspects (presented in “Medical aspects in view of unified cell bioenergetics” section), suggesting practicable possibilities that might assist in disease prevention.
